# Subthalamic deep brain stimulation in Parkinson׳s disease has no significant effect on perceptual timing in the hundreds of milliseconds range

**DOI:** 10.1016/j.neuropsychologia.2014.02.021

**Published:** 2014-05

**Authors:** Thomas E. Cope, Manon Grube, Arnab Mandal, Freya E. Cooper, Una Brechany, David J. Burn, Timothy D. Griffiths

**Affiliations:** a Auditory Group, Institute of Neuroscience, Newcastle University, Newcastle-upon-Tyne, United Kingdom; b Institute for Ageing and Health, Newcastle University, Newcastle-upon-Tyne, United Kingdom

**Keywords:** Perceptual timing, Parkinson׳s disease, Subthalamic nucleus, Deep brain stimulation

## Abstract

Bilateral, high-frequency stimulation of the basal ganglia (STN-DBS) is in widespread use for the treatment of the motor symptoms of Parkinson׳s disease (PD). We present here the first psychophysical investigation of the effect of STN-DBS upon perceptual timing in the hundreds of milliseconds range, with both duration-based (absolute) and beat-based (relative) tasks; 13 patients with PD were assessed with their STN-DBS ‘on’, ‘off’, and then ‘on’ again.

Paired parametric analyses revealed no statistically significant differences for any task according to DBS status. We demonstrate, from the examination of confidence intervals, that any functionally relevant effect of STN-DBS on relative perceptual timing is statistically unlikely. For absolute, duration-based timing, we demonstrate that the activation of STN-DBS may either worsen performance or have no effect, but that it is unlikely to lead to significant improvement.

Although these results are negative they have important implications for our understanding of perceptual timing and its relationship to motor functions within the timing network of the brain. They imply that the mechanisms involved in the perceptual processing of temporal information are likely to be functionally independent from those that underpin movement. Further, they suggest that the connections between STN and the subtantia nigra and globus pallidus are unlikely to be critical to beat-based perceptual timing.

## Highlights

• We assessed the effect of STN-DBS on both duration- and beat-based perceptual timing.
• DBS activation did not profoundly affect beat-based perceptual timing.
• An effect of DBS on duration-based timing was not demonstrated but cannot be excluded.
• Connections between STN and cerebellum are unlikely to be critical to perceptual timing.

## Graphical abstract

Paired difference in performance thresholds according to DBS status. The first and third measures within each triplet show performance for that run with DBS ‘on’ compared to that with DBS ‘off’; values greater than zero denote poorer performance with DBS ‘on’, while those less than zero denote poorer performance with DBS ‘off’. The middle measure shows the difference in performance between the two runs with DBS ‘on’. Error bars denote confidence intervals.

**Figure:**
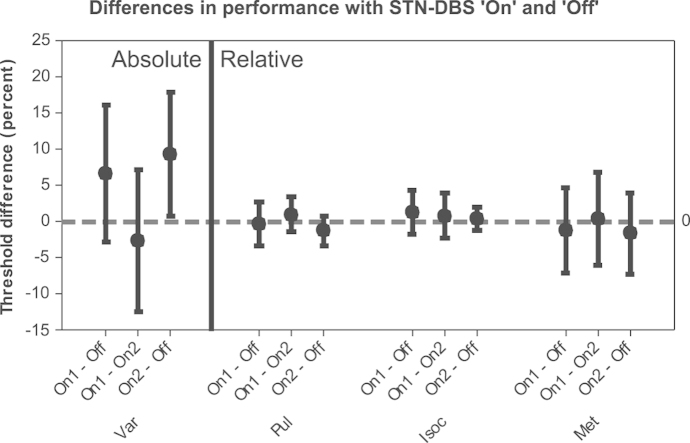

## 1 Introduction

### 1.1 Deep brain stimulation of the subthalamic nucleus (STN-DBS)

#### 1.1.1 DBS as a treatment for Parkinson׳s disease (PD)

Parkinson׳s disease is a degenerative disorder of the central nervous system characterised by the loss of the dopaminergic neurones of the substantia nigra. This results in a clinical syndrome of rigidity, bradykinesia, postural instability, gait disturbance and, often, tremor. Pharmacological therapy focusses primarily on the replacement or modulation of disordered dopaminergic transmission. Bilateral stimulation of the basal ganglia is now a widely implemented treatment for patients with PD whose treatment with dopaminergic therapy is limited by short duration of benefit or dyskinetic side effects (for a review, see (Perlmutter & Mink, 2006)). The main targets for this stimulation are the subthalamic nucleus (STN) (Limousin et al., 1995) and the internal segment of the globus pallidus (GPi) (Pahwa et al., 1997). While DBS of either of these nuclei equally reduces the motor manifestations of PD, STN has become the clinically preferred target as it has been demonstrated to be more likely to allow a reduction in dopaminergic drug use (Anderson, Burchiel, Hogarth, Favre, & Hammerstad, 2005; Rodriguez-Oroz, Matsubara, Clavero, Guridi, & Obeso, 2009). DBS of GPi is now generally reserved for patients with particular difficulties with dystonia (Kern & Kumar, 2007) or mood (Burdick et al., 2011; Okun et al., 2009; Trepanier, Kumar, Lozano, Lang, & Saint-Cyr, 2000). Once a patient with PD has stabilised with long term STN-DBS, changes in stimulator state are rapidly reflected in clinical effect; turning stimulation ‘off’ causes a rapid recrudescence of symptoms, which are quickly relieved by turning it back ‘on’. This is in contrast to the treatment of disorders such as dystonia, where clinical benefit can take many weeks to appear, and symptoms typically reappear slowly once stimulation is stopped.

#### 1.1.2 Cortical and sub-cortical effect of STN-DBS

Despite its widespread clinical uptake, the mechanism of action of DBS is poorly understood. While high-frequency DBS mimics the functional effect of ablation (Breit, Schulz, & Benabid, 2004), there is emerging evidence to suggest that this is achieved through stimulation-induced modulation of brain network activity (Kringelbach, Jenkinson, Owen, & Aziz, 2007). The STN is directly and indirectly anatomically interconnected with a number of brain areas that are involved in the control of movement, cognition and mood (Benarroch, 2008; Hamani, Saint-Cyr, Fraser, Kaplitt, & Lozano, 2004; Nambu, Tokuno, & Takada, 2002). Although the distribution of its connections allows STN to be anatomically segregated into subregions designated *limbic* (medial; behavioural and emotional functions), *associative* (ventromedial; oculomotor and cognitive functions) and *motor* (dorsolateral), these areas are functionally interconnected (Mallet et al., 2007). These properties give STN-DBS the potential to directly affect the function of distributed cortical and sub-cortical networks.

For the initial decades after its introduction, there was reluctance to employ functional magnetic resonance imaging for research purposes in patients with DBS due to concerns about electrode heating and displacement, but since demonstrations of the safe use of this technique (Jech et al., 2001; Rezai et al., 1999) a number of studies have investigated the effects of STN-DBS on regional brain activity. Consistent increases in activation at rest with unilateral STN-DBS have been demonstrated in ipsilateral putamen, thalamus and supplementary motor area, as well as contralateral cerebellum (Phillips et al., 2006; Stefurak et al., 2003). Further, positron emission tomography (PET) studies investigating task-dependant changes in regional cerebral blood flow in patients with PD demonstrated an increase in blood flow to supplementary motor cortex and supplementary motor area during a paced motor task with STN-DBS activated (Ceballos-Baumann et al., 1999).

### 1.2 The neural basis for the perceptual processing of temporal information

The human auditory system is capable of extracting behaviourally relevant temporal features of sound over six orders of magnitude, from the sub-millisecond level relevant to source localisation and pitch processing to the level of seconds and tens of seconds for the processing of sentences and ‘streams’ of sounds (Bregman, 1990; Mauk & Buonomano, 2004). This is performed by hierarchically organised mechanisms, in which longer time windows are processed sequentially further from primary auditory cortex (Cope, Sedley, & Griffiths, 2011; Poeppel, 2003).

The representation of time at and beyond the level of hundreds of milliseconds relies on at least one central mechanism, which is often described as an internal clock (Ivry & Schlerf, 2008; Treisman, 1963). Brain areas implicated in the processing of temporal information at this level include the cerebellum (Ivry, 1993; Ivry & Keele, 1989), basal ganglia (Gibbon, Malapani, Dale, & Gallistel, 1997; Grahn & Brett, 2007; Grahn & Rowe, 2009, 2013; Harrington, Haaland, & Hermanowicz, 1998; Meck & Benson, 2002), supplementary motor area (Halsband, Ito, Tanji, & Freund, 1993; Macar, Anton, Bonnet, & Vidal, 2004) and pre-frontal cortex (Lewis & Miall, 2003, 2006; Oshio, 2011; Oshio, Chiba, & Inase, 2008). The interconnections between these areas have been demonstrated both anatomically (Akkal, Dum, & Strick, 2007; Bostan, Dum, & Strick, 2010; Bostan & Strick, 2010; Hoshi, Tremblay, Feger, Carras, & Strick, 2005; Jurgens, 1984) and functionally, during the performance of timing tasks (Chen, Zatorre, & Penhune, 2006; Grahn & Rowe, 2009).

#### 1.2.1 *Absolute,* duration-based vs *relative,* beat-based timing

While there are undoubtedly further complexities, and room for sub-division, in recent years it has become evident that there is a difference in the neural substrate responsible for the processing of *absolute* time, where the durations of two single intervals are compared, and *relative* time, where comparative judgments of duration are made based on a regular beat (Keele, Nicoletti, Ivry, & Pokorny, 1989; McAuley & Jones, 2003; Teki, Grube, Kumar, & Griffiths, 2011). Perceptual timing performance in normal individuals is superior when *relative*, beat-based information is present (Essens & Povel, 1985; Grube, Cooper, Chinnery, & Griffiths, 2010a; Grube & Griffiths, 2009; Hirsh, Monahan, Grant, & Singh, 1990; Monahan & Hirsh, 1990).

#### 1.2.2 The role of the cerebellum

Recent work supports an obligatory role for the cerebellum in the perceptual processing of *absolute* but not *relative* timing; patients with spinocerebellar ataxia type 6, a stereotyped cerebellar degeneration, were specifically impaired relative to controls when asked to compare the *absolute* duration of discrete intervals (Grube et al., 2010a), a finding consistent with previous investigations of the role of the cerebellum in duration estimation (Harrington, Lee, Boyd, Rapcsak, & Knight, 2004; Ivry & Keele, 1989). This impairment was especially marked when interval duration was roved between trials to minimise overall temporal context, and was absent when a regular beat was present to allow for *relative* comparison. Congruent results have been obtained in a cohort of neurologically normal subjects in whom transcranial magnetic stimulation (TMS) was applied to the medial cerebellum (Grube, Lee, Griffiths, Barker, & Woodruff, 2010b).

#### 1.2.3 The role of the basal ganglia

In functional imaging studies the basal ganglia have been strongly implicated in the analysis of *relative,* beat-based timing (Gibbon et al., 1997; Grahn & Brett, 2007; Grahn & McAuley, 2009; Meck & Benson, 2002; Teki et al., 2011). A physiological mechanism for this function is formulated in the Striatal Beat Frequency model, in which a timing signal is generated by a striato-thalamo-cortical circuit (Meck, Penney, & Pouthas, 2008; Oprisan & Buhusi, 2011). It is proposed that the medium spiny neurons in the dorsal striatum act as coincidence detectors for oscillatory activity (Matell & Meck, 2004; Miall, 1989) and that the periodicity of the oscillator is regulated by dopaminergic input from the substantia nigra (Allman & Meck, 2012; Buhusi & Meck, 2005; Oprisan & Buhusi, 2011).

#### 1.2.4 A unified model of time perception

The ‘unified model of time perception’ proposes that time intervals can be encoded as a combination of a learned, cyclically occurring duration, represented by the basal ganglia through the Striatal Beat Frequency, and an error-correction, supplied by the olivocerebellar network (Teki, Grube, & Griffiths, 2012). This model is supported by recent work demonstrating that patients with intrinsic damage to the striatum (from Huntington׳s disease and Multiple System Atrophy) are significantly impaired in their ability to perform both *absolute* and *relative* perceptual timing tasks (Cope, Grube, Singh, Burn, & Griffiths, 2014).

### 1.3 Timing in Parkinson׳s disease (PD)

While there is generalised neuronal degeneration, the dysfunction in PD is not primarily degeneration of the striatum, but rather an uneven loss of its dopaminergic input (Kish, Shannak, & Hornykiewicz, 1988). Tasks of timing have been extensively assessed in patients with PD (for recent comprehensive reviews see (Allman & Meck, 2012) and (Jones & Jahanshahi, 2014)). When perceptual timing has been assessed in isolation, patients with PD have been found to be impaired on tasks based on a regular beat, but not on those without (Grahn & Brett, 2009). The majority of studies have used tasks with motor confounds, such as tapping out rhythms or reproducing single-interval durations. In general, the findings of the perceptual studies can be attributed to a decrease in the speed of the internal clock (Harrington et al., 1998), especially when medication was withdrawn (Artieda, Pastor, Lacruz, & Obeso, 1992; Jones, Malone, Dirnberger, Edwards, & Jahanshahi, 2008; O׳Boyle, Freeman, & Cody, 1996; Pastor, Artieda, Jahanshahi, & Obeso, 1992). Similar decreases in internal clock speed can be demonstrated in both rats and humans exposed to dopamine blockade, while increases occur with exposure to dopamine agonists (Buhusi & Meck, 2002; Cheng, Hakak, & Meck, 2007; MacDonald & Meck, 2005; Meck, 1983, 1986).

### 1.4 Timing in STN-DBS

The only prior assessment of the effect of STN-DBS on auditory temporal processing was performed by Guehl et al. (2008). They examined the perceptual abilities of patients with PD to detect a gap (of <10 ms) within a constant stimulus, to compare the duration of single intervals against a fixed reference of 50 ms; to detect extra gaps inserted within an otherwise isochronous sequence of clicks presented at 20 Hz; and to detect signals repeated at 2, 16 and 256 Hz within a white noise background. The effects of medication (administered at just under half of patients’ pre-stimulation, therapeutic dose) and STN-DBS on these abilities were assessed. Overall, a statistically significant improvement in performance was demonstrated when the patients were receiving both medication and STN-DBS stimulation compared to when both treatments were withheld, but no significant differences were demonstrated between medication-alone and medication plus STN-DBS. No task by group interaction was demonstrated, but there was a trend for STN-DBS to lead to a more significant improvement in auditory gap detection and deviation from isochrony than in the comparison of single intervals.

While the discrimination of single intervals and detection of gaps in isochronous sequences is conceptually similar to our tasks presented below, Guehl et al. (2008) exclusively assessed perceptual timing below 100 ms, and the isochronous stimulus comprised clicks presented at 20 Hz (an inter-onset-interval of 50 ms). Although the authors interpret their results as evidence against changes in PD of the periodicity of an underlying pacemaker, it is questionable whether such a mechanism would be engaged at these brief timescales. Human sensitivity to changes in duration increases up to around 200 ms, above which the Weber fraction is relatively constant to at least 800 ms (Drake, Botte, & Baruch, 1992; Drake & Botte, 1993; Grondin, 2010; Mauk & Buonomano, 2004). Recent EEG studies elegantly demonstrate that neuronal entrainment to rhythmic stimuli preferentially occurs below 5 Hz, even when the underlying periodicity or metre of the stimulus exceeds this (Nozaradan, Peretz, & Mouraux, 2012).

Koch et al. (2004) tested the effect of STN-DBS on the reproduction of single time intervals of 5 and 15 s. In line with previous work (Malapani et al., 1998), the authors demonstrated that patients with untreated PD consistently over-estimate the shorter interval and under-estimate the longer interval. This effect can be explained by a deviation of subjects’ point of subjective equality towards the overall temporal context of the experiment (McAuley & Miller, 2007; Miller & McAuley, 2005), and reflects a complex interplay of perceptual timing, memory and decision making. The administration of either STN-DBS or dopaminergic medication was sufficient to reduce this effect, but STN-DBS had no further effect on patients who were dopamine-replete. In contrast, Wojtecki et al. (2011) demonstrated that STN-DBS at 10 Hz (therapeutic STN-DBS is usually administered at or above 130 Hz) exacerbated these consistent errors, and replicated the beneficial effect of therapeutic STN-DBS. It is unclear how these results relate directly to the perception of time at the level of hundreds of milliseconds, a temporal region in which patients with PD do not show this “migration” effect (Koch et al., 2008).

STN-DBS has been demonstrated to improve rhythmic motor performance in patients with PD (Joundi, Brittain, Green, Aziz, & Jenkinson, 2012). This study employed the synchronisation-continuation repetitive tapping task (Wing & Kristofferson, 1973), upon which patients with PD have previously demonstrated poor performance (see (Jahanshahi, Jones, Dirnberger, & Frith, 2006) and (Wild-Wall, Willemssen, Falkenstein, & Beste, 2008) for overviews). STN-DBS improved performance almost to the level of control participants, and this was attributed to a reduction in the variance of a central clock generator. These findings, combined with demonstrations that DBS of the internal globus pallidus (Gpi-DBS) improves the precision of internally but not externally timed movements (Schenk, Baur, Steude, & Botzel, 2003), suggest that DBS can be an important regulator of motor timing, and make the examination of its effect on perceptual timing a pressing concern.

### 1.5 Study aims

This study aims to assess the effect of STN-DBS on duration- and beat-based perceptual timing abilities of patients with PD at the level of hundreds of milliseconds, an area of high temporal acuity. As described above, patients with PD are known to be impaired relative to controls on tasks of *relative* timing, consistent with a slowing of the internal clock. These difficulties are reduced, but not eliminated, by dopaminergic replacement. STN-DBS improves the motor symptoms of PD through modulation of brain network activity rather than direct stimulatory input – it is therefore difficult to predict whether it would aid or disrupt basal ganglia perceptual timing functions.

## 2 Methods

### 2.1 Subjects

Thirteen patients with idiopathic PD (4 female), treated with bilateral STN-DBS were recruited at least 6 months after the stabilisation of therapeutic stimulation. All were right handed, and none had any musical training. Demographic and neuropsychometric data are displayed in Table 1, and STN-DBS stimulation parameters are displayed in Table 2. Approval for study conduct was gained from the local research ethics committee and the hospital governance board. All subjects provided informed consent for participation.

### 2.2 Stimuli

All stimuli were constructed from pure tones of 100 ms duration including 20 ms raised cosine onset and offset ramps, with a frequency of 200 Hz and presented at a default volume of 75 dB SPL. If patients reported difficulty in hearing the stimuli clearly or found them uncomfortably loud, they were allowed to adjust the volume of stimulus presentation to their own preference. Stimuli were generated digitally in real time using Matlab 6.5 and Cogent 2000, which was developed by the Cogent 2000 team at the FIL and the ICN and is a toolbox designed for presenting stimuli with precise timing, and presented diotically through external Edirol UA-4X soundcards and Sennheiser HD 250 headphones. Subjects were tested individually in a quiet room.

### 2.3 Procedure

The general procedure for the experiment was as described in (Grube et al., 2010a), except that all subjects undertook each test of perceptual timing three times. Each test was first administered with STN-DBS stimulation “on” at the patients’ usual therapeutic level. STN-DBS stimulation was then turned “off”, and after 5 min without stimulation, subjects undertook the same test again. STN-DBS was thereafter turned “on” again, and after 5 min with stimulation reinstated, subjects undertook the same test a third time. Tests were performed in the order given below. No changes were made to patients’ usual doses of dopaminergic medication.

### 2.4 Tasks

**Fig. 1:**
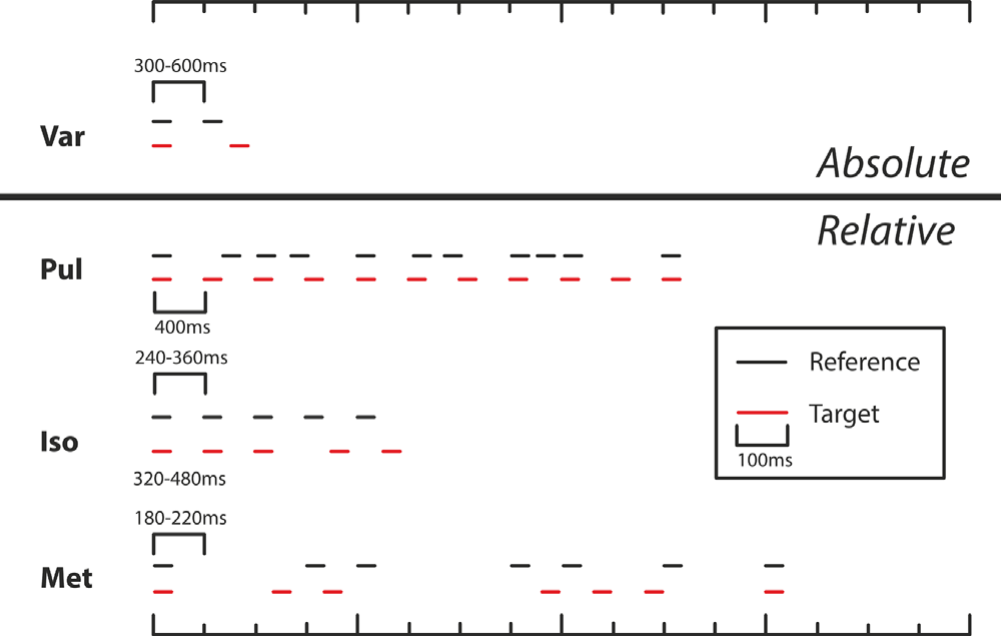
Schematic illustration of stimuli for the tasks of perceptual timing. Horizontal lines depict tones (200 Hz, 100 ms). In each case the reference stimulus is shown above the initial target stimulus. As each run progressed, the target stimulus was adaptively controlled to be increasingly similar to the reference stimulus, dependent on participant performance. (A) Var: sub-second variable-interval discrimination. (B) Pul: detection of regularity (pulse, or beat) within an irregular sequence. (C) Iso: detection of deviation from isochrony. (D) Met: detection of the disruption of a sequence with strong metrical structure.

The tasks are illustrated schematically in Fig. 1. All tasks used an adaptive, two alternative forced-choice procedure to determine perceptual thresholds. Tasks are briefly outlined below, but are described in detail in (Grube et al., 2010a). The only differences to the protocol used by Grube et al. (2010b) were that the fixed-interval timing task (**Fix**) was omitted here, and that additional constraints were placed upon the stimulus generation in the tasks of regular pulse detection such that: the total amount of jitter could never be larger in the target than the reference and was always between 80% and 120% of the intended jitter difference; sequences could not contain more than three shortened or lengthened intervals in a row, or be comprised of primarily shortened or lengthened intervals. To reflect these differences, the short title for this task has been redesignated (**Pul**), from (**Reg)** used by Grube et al. (2010b).

Breaks were provided between tasks, and at the half-way stage (after the second of the four timing tasks) cognitive testing was undertaken. This comprised the Wechsler Test of Adult Reading (WTAR), an assessment of forwards and backwards digit span, and a Revised Addenbrooke׳s Cognitive Examination (ACE-R) (Mioshi, Dawson, Mitchell, Arnold, & Hodges, 2006), from which the fluency component was discarded due to the confound of slowed and impaired speech in patients with PD and STN-DBS.

#### 2.4.1 *Absolute*, duration-based timing

##### 2.4.1.1 Variable interval timing (**Var**)

This task was based on two separated subsecond intervals, and subjects were asked to distinguish a longer target from a shorter reference. Reference inter-onset-interval durations were randomly roved between trials from 300 to 600 ms in 60 ms steps.

#### 2.4.2 *Relative*, beat-based timing

##### 2.4.2.1 Detection of regularity, or pulse, within an irregular sequence (**Pul**)

In this task, subjects were asked to distinguish a target sequence, containing a degree of underlying regularity, from a completely irregular reference sequence. Both sequences consisted of 11 tones presented over 4.5 s and based on a regular 400 ms beat. The reference sequence was rendered highly irregular by randomly shortening or lengthening each interval by an average of 30% (range 15–45%), making the underlying beat imperceptible (Madison & Merker, 2002). A similar procedure was applied to the target sequence, but the mean jitter in this sequence began at 0% (perfect isochrony). During the task, this value was adaptively controlled in steps of 4% for the first four turnpoints and 2.5% thereafter, but could never exceed the jitter of the reference.

##### 2.4.2.2 Detection of deviation from isochrony (**Iso**)

This task used a 5-tone isochronous reference sequence with a 300 ms inter-onset interval, against which subjects had to discriminate a target sequence with a lengthening of the third inter-onset interval (preceding the 4th tone).

##### 2.4.2.3 Detection of disruption of a metrical pattern (**Met**)

In this task, subjects were asked to identify a target sequence in which the rhythmic pattern of a strongly metrical, seven-tone sequence was disrupted. The sequence was based upon two levels of periodicity; a lower level periodicity of 180–220 ms and a higher level metrical beat of four, formed by the regular occurrence of temporally induced accents on every fourth beat (every 800 ms on average) (Grube & Griffiths, 2009; Povel & Essens, 1985). The target sequence was adaptively disrupted by a balanced change in the length of the longer silent intervals (two were lengthened and two were shortened by an equal proportion), while the shorter silent intervals were unchanged. The amount of distortion was adaptively controlled, starting at 65% and changed in steps of 12% for the first four turnpoints and 6% thereafter.

### 2.5 Statistical analysis

Patients acted as their own controls, with all analyses based on paired comparisons between their performance on each of the three repetitions of each test. The distribution of these data was explored in IBM SPSS Version 19 by the Kolmogorov-Smirnov test with Lillefors significance correction; performance differences did not deviate significantly from a normal distribution. Mauchly׳s test of sphericity confirmed that variance could be assumed to be equal between comparison pairs. Differences were therefore suitable for assessment with quantitative statistics, and specifically by analysis of variance (ANOVA).

## 3 Results

**Fig. 2:**
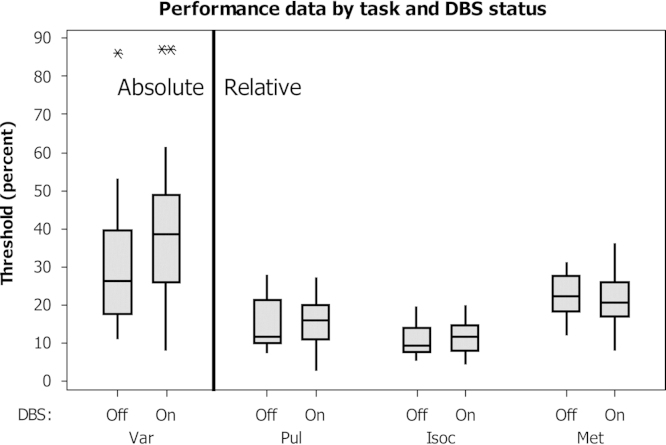
Illustrative boxplots of measured thresholds by task and DBS status. Box denotes inter-quartile range, central line denotes median, whiskers describe the range of all measures within two standard deviations, and asterisks denote outliers. Threshold is expressed as a percentage difference in inter-onset interval for tasks Var, Isoc and Met, and as percentage jitter difference for task Pul (the reference sequence always had 30% jitter, so the absolute jitter value at which regularity could be detected can be derived by subtracting the threshold presented from 30). Higher values denote poorer performance.

Threshold performance was measured by adaptive tracking of thresholds for all four perceptual timing tasks (Var, Reg, Iso, Met) with DBS ‘on’ (On1), ‘off’ (Off) and then ‘on’ again (On2). Illustrative boxplots for the group threshold data are displayed in Fig. 2. Supplementary Fig. S1 illustrates the patient threshold data compared to a group of age-matched, healthy control participants – these data are briefly discussed in appendix A.

**Fig. 3:**
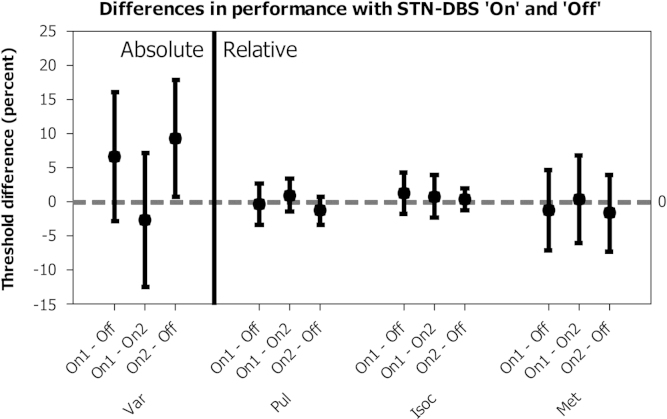
Paired difference in performance thresholds by DBS status, expressed as percentage of inter-onset-interval for tasks Var, Isoc and Met, and percentage jitter for Pul. The first and third measures within each triplet show performance for each run with DBS ‘on’ (run 1&3) compared to that with DBS ‘off’ (run 2); values greater than zero denote a deterioration in performance with DBS ‘on’ compared to ‘off’, while those less than zero denote an improvement in performance in ‘on’ compared to ‘off’. The middle measure shows the difference in performance between the two runs with DBS ‘on’. Error bars denote confidence interval, and symbols the group mean.

Each subject׳s threshold in each of these conditions was compared to assess any consistent difference in performance according to DBS status (Fig. 3). If there were a consistent improvement in performance with DBS ‘on’ we would expect both the first and third measures (On1 – Off) and (On2 – Off) to be below zero, while a consistent deterioration in performance would result in them being above zero. In each case, the middle measurement (On1 – On2) should still approximate to zero, unless there were a significant effect of practice, fatigue or the timing of testing relative to medication doses. All confidence intervals for the means of differences include zero, except the third measure (On2 – Off) in test Var, which extends from 0.68% to 18%. A balanced ANOVA of differences demonstrated no significant effect of DBS status (*p*=0.569) or test (*p*=0.084). Although there was no prior hypothesis for a differential effect of DBS on test Var, a confirmatory one way ANOVA was performed for this condition, which again demonstrated no significant difference according to DBS status *p*=0.128).

**Fig. 4:**
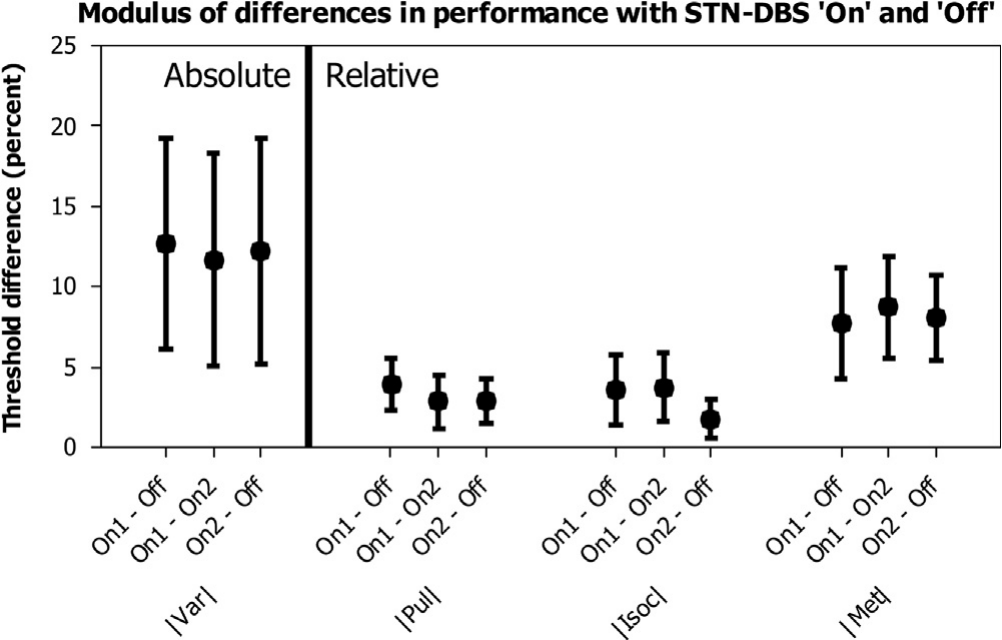
Paired absolute differences in performance thresholds by DBS status. The first and third measures within each triplet show performance with each run with DBS ‘on’ compared to that with DBS ‘off’, while the middle measure shows the difference in performance between the two runs with DBS ‘on’. Higher values denote greater change between runs, regardless of whether this change is improvement or deterioration of performance. Error bars denote confidence interval, and symbols the group mean.

Because of the widespread cortical projection of the STN, and the inevitable variation in electrode position within this small structure, it was hypothesised that DBS might impact upon perceptual timing in an inconsistent way between subjects (i.e. some individuals might improve and others get worse). In order to investigate this, the absolute (unsigned) difference in thresholds was calculated between each run pair for each task (Fig. 4). The (On1 – On2) comparison was again included for comparison as a measure of inter-run variability due to practice or fatigue. In this figure, if DBS significantly impacted upon performance in an inconsistent manner, the first (On1 – Off) and third (On2 – Off) values would both be significantly greater than the second (On1 – On2) for the affected task. Four of these values follow this pattern and four do not, and none of the differences are large. Confidence intervals for all (On1 – On2) comparisons encompass the means of all (On – Off) comparisons for each test and no statistically significant differences were found on one way ANOVAs performed for each test individually.

## 4 Discussion

### 4.1 The lack of an effect of STN-DBS: absence of evidence or evidence of absence?

No significant difference in the performance of any task was found when STN-DBS was turned ‘off’, nor when it was turned back ‘on’. This could be simply interpreted as a lack of influence of STN-DBS on perceptual timing, especially as the same or similar perceptual tasks have previously proven sufficiently sensitive to detect perceptual timing difficulties in patients with cerebellar disease or inactivation (Grube et al., 2010a, 2010b) and basal ganglia disease (Cope et al., 2014), without the statistical benefit of within-subject comparison. It is a basic tenant of statistical theory, however, that absence of evidence is not evidence of absence. One might therefore argue that the inclusion of a large enough number of patients might increase statistical power sufficiently to find statistically significant differences. It is impossible to demonstrate from our data that this is not the case, but it is possible to demonstrate that the magnitude of this difference would be unlikely to be functionally relevant, through the examination of confidence intervals (Blackwelder, 1982; Streiner, 2003).

For the detection of regularity within an irregular sequence (Pul), the largest differences in thresholds described by the 95% confidence intervals in Fig. 3 are 2.6% and −3.5%. With STN-DBS ‘on’, the group median threshold indicated that patients were able to detect the underlying regular beat in the presence of a jitter of 14.3%. In other words, turning ‘off’ STN-DBS would at best improve performance such that, on average, patients could detect regularity in a sequence with up to 16.9% jitter, and at worst impair it so that regularity was only detectable in the presence of up to 10.8% jitter. Similarly for task Iso, turning ‘off’ STN-DBS would at best reduce the minimum amount of deviation from isochrony required for its detection from 11.6% to 9.8%, and at worst increase it to 16.4%. Finally, for the task based upon metrical rhythms (Met), turning ‘off’ STN-DBS would at best reduce the minimum detectable amount of sequence disruption from 20.5% to 16.9% and at worst increase it to 27.5%. Although any consistent effect upon relative, beat-based timing thresholds would arguably be of scientific interest, we would attest that these potential changes are sufficiently small to conclude that bilateral disruption of the subthalamic nucleus with deep brain stimulation does not have a profound effect upon the performance of perceptual tasks of relative timing.

Such a clear conclusion cannot be reached for the perceptual task of absolute timing employed here. Although no statistically significant difference was demonstrated, the confidence intervals indicate that turning STN-DBS ‘off’ could have improved average thresholds by as much as 17.9%, while it is statistically unlikely that it worsened them by more than 2.8%. In other words, it is statistically plausible that the median minimum detectable difference in the length of two single intervals could improve from 39.5% in patients with PD with STN-DBS turned ‘on’ to 21.6% in the same patients with STN-DBS turned ‘off’. This would represent almost a halving of temporal acuity due to the introduction of STN-DBS. Similarly, it is statistically plausible that the STN-DBS had no effect, or even that it could slightly improve performance, as the worst plausible ‘off’ threshold was 42.3%. Guehl et al. (2008) also demonstrated no change in performance in the timing of single intervals with STN-DBS state, but it is difficult to compare these data to ours as the intervals tested were an order of magnitude more brief, and the duration of the comparison interval was fixed at 50 ms, rather than being roved from trial to trial. We can therefore draw no strong conclusions about the effect of STN-DBS on perceptual tasks of absolute timing, but have to consider the possibility of a deterioration in performance as a function of stimulation being switched “on”.

### 4.2 Methodological limitations

When a study demonstrates the absence of differences between groups, it is important to consider whether there are any methodological limitations that could have impaired the sensitivity of the paradigm employed. We discuss, in the following, a number of possible limitations in this study, and their potential impact upon the data presented.

#### 4.2.1 Task sensitivity

From the results presented in this manuscript alone, it might be possible to draw the conclusion that the tasks employed were generally insensitive to performance differences between groups, or that they might have been performed at ceiling or floor level by both groups. It is, however, unlikely that this is the case because our laboratory have demonstrated differences between groups on all of the tasks at performance levels both better (young adults) and substantially worse (patients with Huntington׳s disease) than the data presented here. Grube et al. (2010a) demonstrated poorer absolute but not relative task performance in a group of patients with spinocerebellar ataxia type 6 compared to a matched control group. Cope et al. (2014) demonstrated that patients with Huntington׳s disease performed more poorly than patients with Multiple System Atrophy, who again performed more poorly than controls in tasks Var, Iso and Met. No group difference was demonstrated in task Pul. Grube, Cooper, and Griffiths (2013) in turn demonstrated strong correlations between performance in task Pul and literacy skills in early adulthood.

#### 4.2.2 Interval between ‘On’ and ‘Off’ states

In this study, patients performed each task three times in a row, i.e. with STN-DBS ‘On’, ‘Off’, and then ‘On’ again, before moving on to the next task. The break between a change in STN-DBS state and the start of the next test was 5 min each time. This is sufficient time for the recrudescence or dissipation of motor symptoms, and the rationale for minimising this time was to minimise patient discomfort and to allow repetitions of the same task to be undertaken with as little change as possible in drug levels, fatigue or other factors. The success of our approach to dealing with these potential problems is evident from the finding that there were no systematic differences in performance between the first and third run on any of the tasks (Fig. 2).

Although the clinical effect of STN-DBS in PD is rapid, with initial onset over seconds to minutes, this is not the case in some of the other disorders for which DBS is employed such as dystonia, where clinical improvement is typically observed after 4–6 weeks of stimulation of the Globus Pallidus interna (GPi) and can continue for up to 6 months (Kupsch et al., 2006; Ostrem, Marks, Volz, Heath, & Starr, 2007). Cases have been reported where, after several years of stimulation, sustained relief from dystonia has been obtained when stimulation is withdrawn (Goto & Yamada, 2004; Hebb, Chiasson, Lang, Brownstone, & Mendez, 2007). From these observations, and evidence from direct neurophysiological recordings, it has become evident that DBS has a longer-term, neuromodulatory effect over weeks to months (Ruge et al., 2011; van Hartevelt et al., 2014). It is possible that these longer-term effects might modulate perceptual timing, and that these effects would have been missed by our paradigm. The examination of such effects would be most feasible as a longitudinal study.

### 4.3 Anatomical implications

**Fig. 5:**
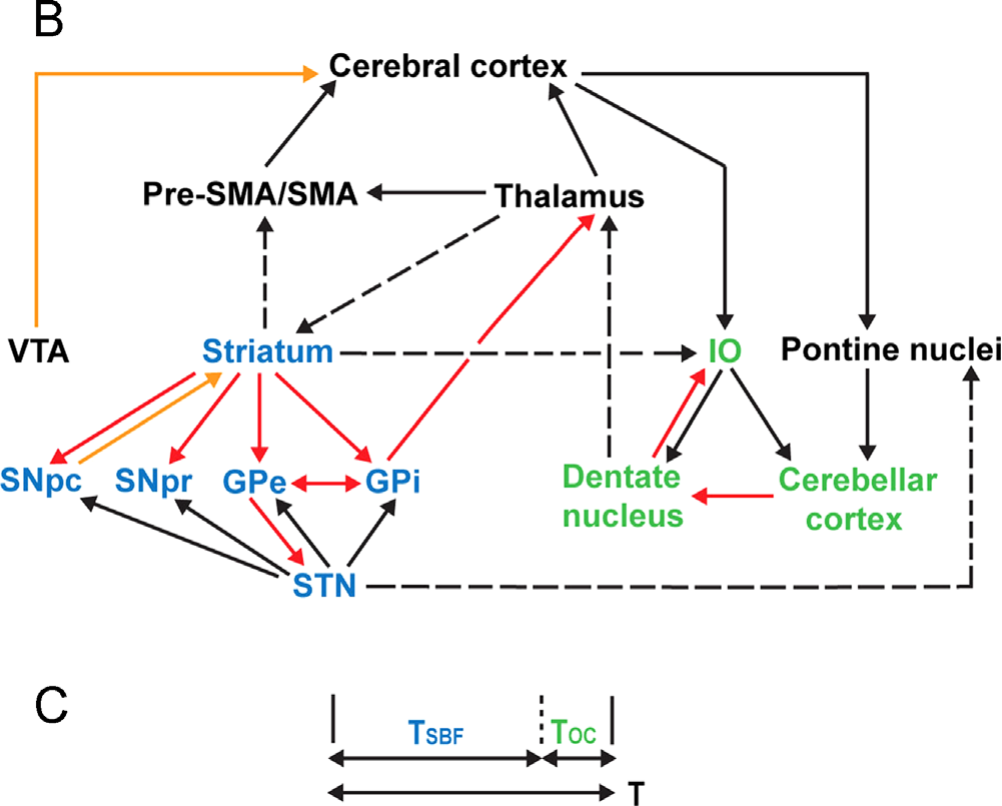
The unified model of time perception, reproduced with permission from (Teki et al., 2012). Part A of the original figure is omitted. Part B of the figure illustrates the striatal network in blue, and the cerebellar network in green. These networks are connected to each other by multiple loops, as well as to the cerebral cortex, SMA, pre-SMA and thalamus. Part C illustrates the proposed mechanism of the unified model, with an estimate of an overall time interval, T, provided by the striatal network in the form of the Striatal Beat Frequency (T_SBF_), with an error correction provided by the olivocerebellar network (T_oc_). Key: orange arrows represent dopaminergic pathways, red arrows represent inhibitory projections, black arrows represent excitatory projections, and dashed arrows represent known anatomical connections. IO=inferior olive; GPe=external globus pallidus; GPi=internal globus pallidus; SNpc=substantia nigra pars compacta; SNpr=substantia nigra pars reticulata; STN=subthalamic nucleus; VTA=ventral tegmental area. (For interpretation of the references to color in this figure legend, the reader is referred to the web version of this article.)

The ‘unified model of time perception’ proposes that time intervals can be encoded as a combination of a learned duration, supplied by the basal ganglia through the Striatal Best Frequency, and an error-correction, supplied by the olivocerebellar network (Teki et al., 2012). The proposed anatomical basis for this model is illustrated in Fig. 5. The basal ganglia and cerebellum are interconnected by a number of direct and indirect pathways (Bostan et al., 2010; Bostan, Dum, & Strick, 2013; Bostan & Strick, 2010), including through a disynaptic pathway from STN (Bostan et al., 2010). Previously published behavioural work implicates an obligatory role for the cerebellum in absolute, but not relative, timing (Grube et al., 2010a, 2010b) and for the basal ganglia in both absolute and relative timing tasks (Cope et al., 2014). We propose that the present behavioural findings demonstrate that the connections between STN and the subtantia nigra and globus pallidus are not critical to the functional role of the basal ganglia in providing a ׳pacemaker׳, with a particular role in perceptual timing.

Instead, it seems likely that perceptual timing is subserved by a ‘core’ functional network involving the striatum, olivo-cerebellar system (Xu, Liu, Ashe, & Bushara, 2006) and supplementary motor area (Kotz & Schwartze, 2011; Schwartze, Rothermich, & Kotz, 2012) that interact through their known direct anatomical connections. One can speculate a role for indirect connections through the STN and thalamus in the integration of independent processing loops to allow, for example, the co-ordination of timed movements.

## 5 Conclusions

The behavioural work presented here demonstrates that STN-DBS is statistically unlikely to have a functionally significant effect upon relative, beat-based, timing. No firm conclusions can be drawn about the effect of STN-DBS on absolute, duration-based, timing, other than that its presence has either no effect or worsens performance. Although negative results are often viewed with disappointment by the scientific community, these findings have important implications for anatomical models of perceptual timing. Specifically, the present findings indicate that the oscillatory loops that are thought to underpin movement (STN-GPe) and perceptual timing (striatum-thalamus-cortex) are likely to be functionally independent. Further, they imply that the disynaptic connection between STN and cerebellum is unlikely to be critical to relative perceptual timing, but leave open the possibility of a specific role for this connection in the analysis of absolute, duration-based time intervals. Further, they indicate that the oscillatory loops that underpin movement (STN-GPe) and perceptual timing (striatum-thalamus-cortex) are likely to be functionally independent. Future research directions might investigate the role of other anatomical connections between cerebellum and basal ganglia, perhaps in the first instance by the observation of perceptual timing performance in patients with GPi-DBS for dystonia or thalamic DBS for essential tremor.

## Figures and Tables

**Table 1:** Demographic and neuropsychometric data for patients with PD and bilateral STN-DBS. Pre-morbid full scale IQ (PMFSIQ) was estimated with the Wechsler Test of Adult Reading (WTAR), which has been demonstrated to give comparable results in PD and healthy controls (Dalrymple-Alford et al., 2011). MMSE=Mini mental state examination. ACE-R=Addenbrooke׳s cognitive examination, revised edition. The fluency component has been excluded from the ACE-R, resulting in a maximum score of 86, because of the significant slowing of speech in patients with PD and STN-DBS.

Subject number	Age	Sex	Years since PD diagnosis	Years since STN-DBS implanted	Estimated PMFSIQ	MMSE (max 30)	ACE-R minus fluency (max 86)
1	55	M	10	7	92	30	81
2	55	M	11	4	113	30	82
3	51	M	9	2	106	29	80
4	56	M	10	3	120	30	86
5	50	F	5	2	104	30	78
6	75	F	8	5	108	30	77
7	64	M	16	7	123	29	82
8	57	M	15	5	108	30	86
9	57	M	29	2	96	29	77
10	64	M	11	3	97	30	76
11	72	F	21	2	90	30	79
12	65	M	15	4	103	29	79
13	61	F	23	4	106	30	82
Median	57		11	4	106	30	80

**Table 2:** STN-DBS stimulation parameters for patients with PD. Stimulation frequency is expressed in Hertz, stimulation amplitude in volts, and pulse width in microseconds.

Subject number	Left			Right		
	Stimulation frequency	Stimulation amplitude	Pulse width	Stimulation frequency	Stimulation amplitude	Pulse width
1	160	3.1	90	160	5.2	60
2	130	4.0	90	130	4.0	60
3	145	5.0	120	145	4.0	90
4	130	3.8	60	130	3.8	60
5	130	4.1	60	130	4.1	60
6	170	3.3	60	170	2.0	90
7	137	3.3	60	137	3.0	60
8	155	4.9	60	155	4.2	60
9	130	3.8	60	130	3.9	60
10	157	3.3	90	157	3.9	60
11	130	2.4	60	130	2.9	60
12	130	3.7	90	130	3.9	90
13	130	3.9	60	130	3.8	60
