# Supplementary material for: Subthalamic deep brain stimulation in Parkinson׳s disease has no significant effect on perceptual timing in the hundreds of milliseconds range
Source: Neuropsychologia. 2014 May;57(100):29–37. doi: 10.1016/j.neuropsychologia.2014.02.021 (PMC4022837; doi:10.1016/j.neuropsychologia.2014.02.021)
Supplement: Supplementary file 1 — Supplementary Material [file mmc1.doc]

**Appendix 1: Comparison of patient data with healthy control subjects**

In this appendix, we briefly describe the comparison of the patient thresholds presented in the main body of the manuscript to those of a group of healthy control participants.

**Subjects:**

Fourteen individuals with no neurological disease were recruited through open advertisement and each paid £20 for their time. None had ever previously participated in an auditory psychophysics experiment. They comprised nine males and five females. One was left handed. They were matched to the patients in age (mean 55 years, range 43 – 74 years, t-test for difference between group means: p=0.15).

**Methods:**

All control participants completed each task three times, so that they had the same amount of exposure to the paradigm as the patients. Threshold was calculated as mean performance over these three runs.

**Statistical analysis:**

Unlike the paired difference data presented in the main body of the manuscript, which were normally distributed, all absolute threshold datasets violated the assumption of normality by either Kolmogorov-Smirnov (Var, Pul, Isoc) or Shapiro-Wilk (Var, Isoc, Met) analyses. It was possible to normalise the threshold datasets for tasks Var, Isoc and Met by taking the natural logarithm of the data, but task Pul still violated this assumption. Conversely, datasets for tasks Pul, Isoc and Met were normalised by taking the square root of the threshold, but in this case task Var violated the assumption of normality. Kolmogorov-Smirnov test statistics were lowest for natural logarithms of threshold for tasks Var and Isoc, and for square root of threshold for tasks Pul and Met.

Group differences were assessed for each test individually in Minitab 16 with repeated-measures, mixed-methods, nested general linear model comparisons (Laird & Ware, 1982), with the individual identifier of ‘subject number’ included as a random and nesting factor. The main effects of group and ‘subject number’ were assessed.

**Results:**

Task thresholds by group are shown in supplementary figure 1, and the results of statistical analyses described above are presented in supplementary table 1.

| **Test** | **Normalisation technique** | **Group** | **Subject number** |
| --- | --- | --- | --- |
| **Var** | Natural logarithm | **<0.001** | **0.003** |
| **Var** | Square root | Not appropriate | Not appropriate |
| **Pul** | Natural logarithm | Not appropriate | Not appropriate |
| **Pul** | Square root | 0.064 | **<0.001** |
| **Isoc** | Natural logarithm | **0.050** | **0.008** |
| **Isoc** | Square root | **0.038** | **0.005** |
| **Met** | Natural logarithm | 0.077 | 0.072 |
| **Met** | Square root | 0.066 | **0.047** |

**Supplementary table 1: Results of the repeated-measures, mixed-methods, nested general linear model comparison between groups (p values; red and bold, significant effect).**

Group performance differences were demonstrated for tasks Var and Isoc, and in both cases the greatest difference was between controls and patients when their DBS was ‘on’. For tasks Pul and Met these comparisons approached, but did not reach, statistical significance.

There was a significant effect of subject number for all tasks in at least one analysis, indicating that, overall, repeated measures for each subject was consistent within tasks.

**Discussion:**

These data are presented only to give a general indication to the interested reader of the performance of patients with Parkinson’s disease on the tests of perceptual timing we present in this study relative to healthy controls of a similar age. The broad trend is for the patients to perform more poorly than the control participants on all of the tasks. This difference reached two-tailed statistical significance for tasks Var and Isoc, while one-tailed significance would be reached for tasks Pul and Met. It is notable that the magnitudes of these group differences are much smaller than those demonstrated using a similar paradigm to compare control patients peripheral neuropathies to those with multiple system atrophy and Huntington’s disease (Cope et al., 2013).

We do not wish the statistically significant results presented here to overshadow the main aim of this study, which was to assess the impact of STN-DBS on perceptual timing. As such, for the main study, the patients acted as their own controls, and the meaningful comparison is made in the same individuals, with all other parameters held the same, between DBS ‘on’ and DBS ‘off’ states. The inclusion of a control group without neurological disease provides some indication of the impact of idiopathic Parkinson’s disease on perceptual timing, but provides no information at all about the effect of STN-DBS.

While we hope that these data help the reader to contextualise our main findings, we do not claim that they represent a robust and comprehensive assessment of timing in Parkinson’s disease, not least because the groups were matched in age and gender, but not in exposure to neurological services, motivation to participate, education or neuropsychometric performance. For recent detailed reviews and discussion of the extensive literature of timing in Parkinson’s disease, we would refer the reader to Allman and Meck (2012), and Jones and Jahanshahi (2014).

Figure legend:

**Supplementary figure 1: Threshold values by group, expressed as a percentage of inter-onset-interval for tasks Var, Isoc and Met, and percentage jitter difference for task Pul (the reference sequence always had 30% jitter, so the absolute jitter value at which regularity could be detected can be derived by subtracting the threshold presented from 30). Error bars denote 95% confidence intervals for the mean for each group. Control values represent the mean of all three repetitions of each task. Patient values with “DBS On” represent the mean of the first and third repetition of each task, while those with “DBS Off” are the mean of the second repetition of each task.**
