# Supplementary figures and images for: Subthalamic deep brain stimulation in Parkinson׳s disease has no significant effect on perceptual timing in the hundreds of milliseconds range
Source: Neuropsychologia. 2014 May;57(100):29–37. doi: 10.1016/j.neuropsychologia.2014.02.021 (PMC4022837; doi:10.1016/j.neuropsychologia.2014.02.021)

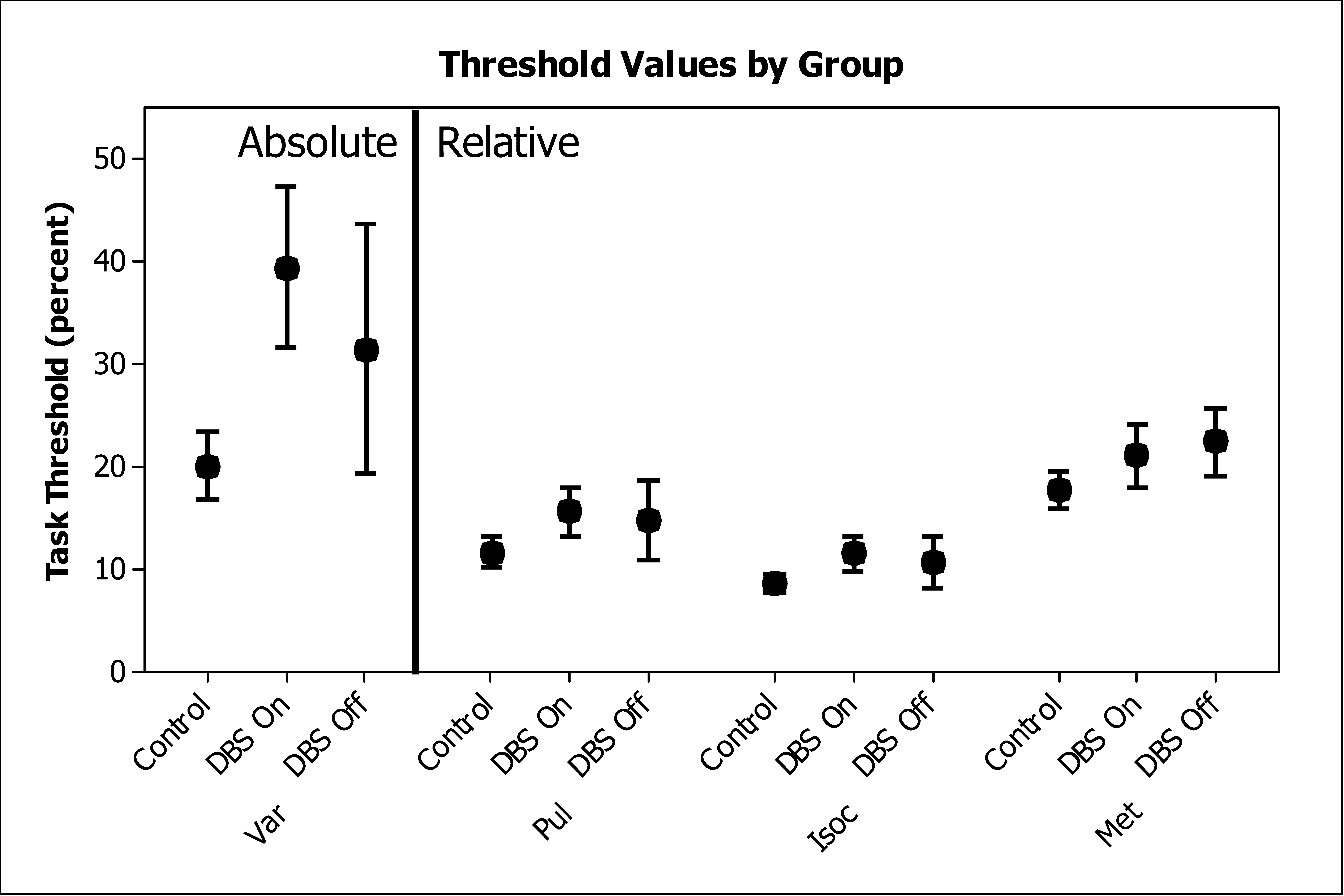

Supplement: Supplementary file 2 — Supplementary Material [file mmc2.zip › Supplementary figure 1.png]
